# PM10 and PM2.5 real-time prediction models using an interpolated convolutional neural network

**DOI:** 10.1038/s41598-021-91253-9

**Published:** 2021-06-07

**Authors:** Sangwon Chae, Joonhyeok Shin, Sungjun Kwon, Sangmok Lee, Sungwon Kang, Donghyun Lee

**Affiliations:** 1 Department of Business Administration, Korea Polytechnic University, 237 Sangidaehak-ro, Siheung-si, 15073 Gyeonggi-do Republic of Korea; 2 Korea Environment Institute, 370, Sicheong-daero, Sejong-si, 30147 Republic of Korea

**Keywords:** Environmental sciences, Engineering

## Abstract

In this paper, we propose a real-time prediction model that can respond to particulate matters (PM) in the air, which are an indication of poor air quality. The model applies interpolation to air quality and weather data and then uses a Convolutional Neural Network (CNN) to predict PM concentrations. The interpolation transforms the irregular spatial data into an equally spaced grid, which the model requires. This combination creates the interpolated CNN (ICNN) model that we use to predict PM10 and PM2.5 concentrations. The PM10 and PM2.5 evaluation results show an effective prediction performance with an R-squared higher than 0.97 and a root mean square error (RMSE) of approximately 16% of the standard deviation. Furthermore, both PM10 and PM2.5 prediction models forecast high concentrations with high reliability, with a probability of detection higher than 0.90 and a critical success index exceeding 0.85. The proposed ICNN prediction model achieves a high prediction performance using spatio-temporal information and presents a new direction in the prediction field.

## Introduction

In Korea and across East Asia, emissions due to rapid economic growth are causing complex and extensive air pollution problems^1^. Particulate matter (PM) is the main cause of air pollution and has been linked to the development of lung cancer^2,3^ as well as respiratory^4,5^, cardiovascular^6,7^, and cerebrovascular diseases^8^. One epidemiological study reported that long-term exposure to high concentrations of PM could raise mortality by as much as 5%^9^. Air pollution caused by PM has been shown to significantly impact both the mental and physical health of a population^10,11^. Therefore, monitoring and predicting the PM concentration in the air is critical to providing early warnings to residents and to helping governments take timely actions^12^. Further, since reliable information on PM concentrations can be used for public health purposes, prediction models capable of accurately forecasting high concentrations of PM are needed^13^.

There are two main types of PM prediction models: physics-based and data-driven. Physics-based models employ the basic principles of atmospheric chemistry and physics^14^. Examples of physics-based models include models using nonlinear empirical models^15,16^ and models combining regression and cellular automata(CA)^17^. However, physics-based models are not as accurate as data-driven models, due to the complex, dynamic nature of air pollution and the uncertainty within these models^15–18^. Also, physics-based models cannot include long-term and short-term features at the same time^15–18^. Data-driven models quantify the complex relationships between air pollutants and potential predictors based on data collected for various atmospheric conditions. Examples of data-driven models include those using statistics-^19,20^ and machine learning-based methodology^21–26^. However, if the data-driven model is used alone when predicting PM, spatial information cannot be easily used due to the characteristics of certain models^18^. The spatial changes of PM are associated with the complex interplay of many parameters, including temperature^27^ , precipitation^28^, wind^29^, and other pollutants such as nitrate oxides (NO)^30^. Moreover, parameters for different locations present different spatial distributions. For this reason, some studies have integrated spatial diversity to reduce measurement errors and improve statistical capacity^31,32^. Success in this area suggests that applying integrated spatial information to the data-driven model could lead to a high-performance PM prediction model.

To consider spatial information in some social phenomena, models using convolutional neural networks (CNN) have been proposed. The predictive powers of CNN models reflecting spatial information have been reported to be high^33–35^. However, it would be difficult to directly integrate spatial information and apply it to a CNN prediction model for PM; the locations of stations for monitoring PM are different from those for monitoring the relevant interacting parameters. Consequently, this paper proposes an interpolated convolutional neural network (ICNN) model for predictions of PM pollution for South Korea that integrates the spatial diversity of the parameters related to PM, the air pollution predictions for areas with no monitoring stations, and the air pollution of individual areas.

In our study, we found that some areas had no air pollution monitoring stations and existing monitoring stations were unevenly spaced. These stations are concentrated in densely populated downtown areas, which has led to limited measurements and forecasts of PM in suburban areas. We addressed this problem by dividing South Korea using an evenly spaced grid and creating virtual monitoring stations through interpolation. This allowed us to interpolate a small set of actual data concentrated in downtown areas to the entire area, thus enabling predictions for places where the air quality is unknown. Moreover, we designed a model based on spatial information for predicting pollution in multiple areas using the spatial characteristics of the interpolated data.

The contributions of this study are as follows. First, the ICNN prediction model has high PM prediction accuracy and simultaneously predicts PM concentration of large areas, including unmonitored areas, by learning spatio-temporal information from big data. Second, we can learn effectively with the ICNN prediction model by transforming the non-uniformly spaced data measured by monitoring stations in different locations into uniformly distributed spatial data. Therefore, ICNN can be used for predicting not only PM but also various environmental fields using spatio-temporal data.

## Approach

### Inverse distance weighting (IDW)

Inverse distance weighting (IDW) interpolation is one of the most widely used spatial interpolation methods^36^. It can create estimates for locations without data, based on data at nearby locations. The advantages of IDW interpolation include its ease of use and fast interpolation process^37,38^. In this study, we use this method in order to interpolate missing values and generate grid-shaped data in ICNN prediction models.

### Convolutional neural networks (CNN)

CNN is an algorithm based on a hierarchical neural network designed to process multidimensional array data. When the CNN receives multidimensional array data, an array of weights called a “convolutional filter” operates on the input array and passes through a nonlinear function to produce the final output^39^. In this study, the CNN model was used to train and predict data created in the interpolating process of the ICNN prediction model.

### Interpolated convolutional neural network (ICNN)

**Figure 1:**
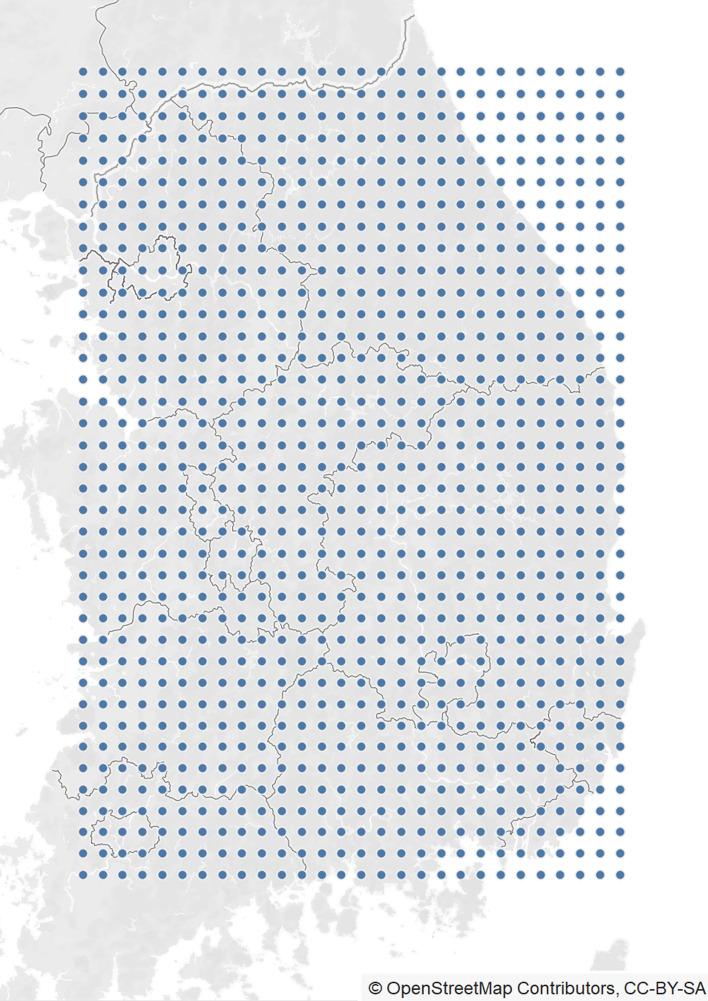
Locations of gridlines for ICNN. Map image is obtained from OpenStreetMap (openstreetmap.org) and licensed under CC-BY-SA (https://www.openstreetmap.org/copyright).

We attempted to convert the air quality monitoring station data into multidimensional arrays. However, the air pollution monitoring stations in South Korea are concentrated in specific regions, and the geographical distances between the stations are unequal. This indicates that the spatial data of the measured values are not uniform. Hence, we propose an ICNN prediction model that performs data transformation and training in turn. The ICNN prediction model transforms the spatially imbalanced measured values into uniform data through interpolation and then predicts the PM concentration through a CNN model. To solve the spatial imbalance, the ICNN prediction model creates an equally spaced empty grid. Subsequently, equal distance data are generated by interpolating the data as if a virtual measuring station were located at each grid point as shown in Fig. 1.

The generated equal distance data are used as input data of a CNN model that performs training within the ICNN prediction models. The ICNN prediction model learns the properties of the data by using pre-interpolated data in the form of a grid during model training, which is expected to increase the prediction accuracy.

### Ordinary least squares (OLS)

Ordinary least squares (OLS) is a simple linear regression approach, which is commonly used in social science research^40,41^. It is used to find the parameter that minimizes the sum of the squared errors. In this study, the OLS regression method was used as a comparative model to evaluate the performance of the ICNN prediction model.

### Long-short term memory (LSTM)

Long short-term memory (LSTM) is a commonly used model for sequential data processing, such as voice or text processing^42^. The LSTM model has been used recently for predictions based on time series data^18,24^. It is also combined with other methods to form a new predictive model^43,44^. In this study, the LSTM model was used as a comparative model for the ICNN prediction model.

## Evaluation

### Dataset

**Figure 2:**
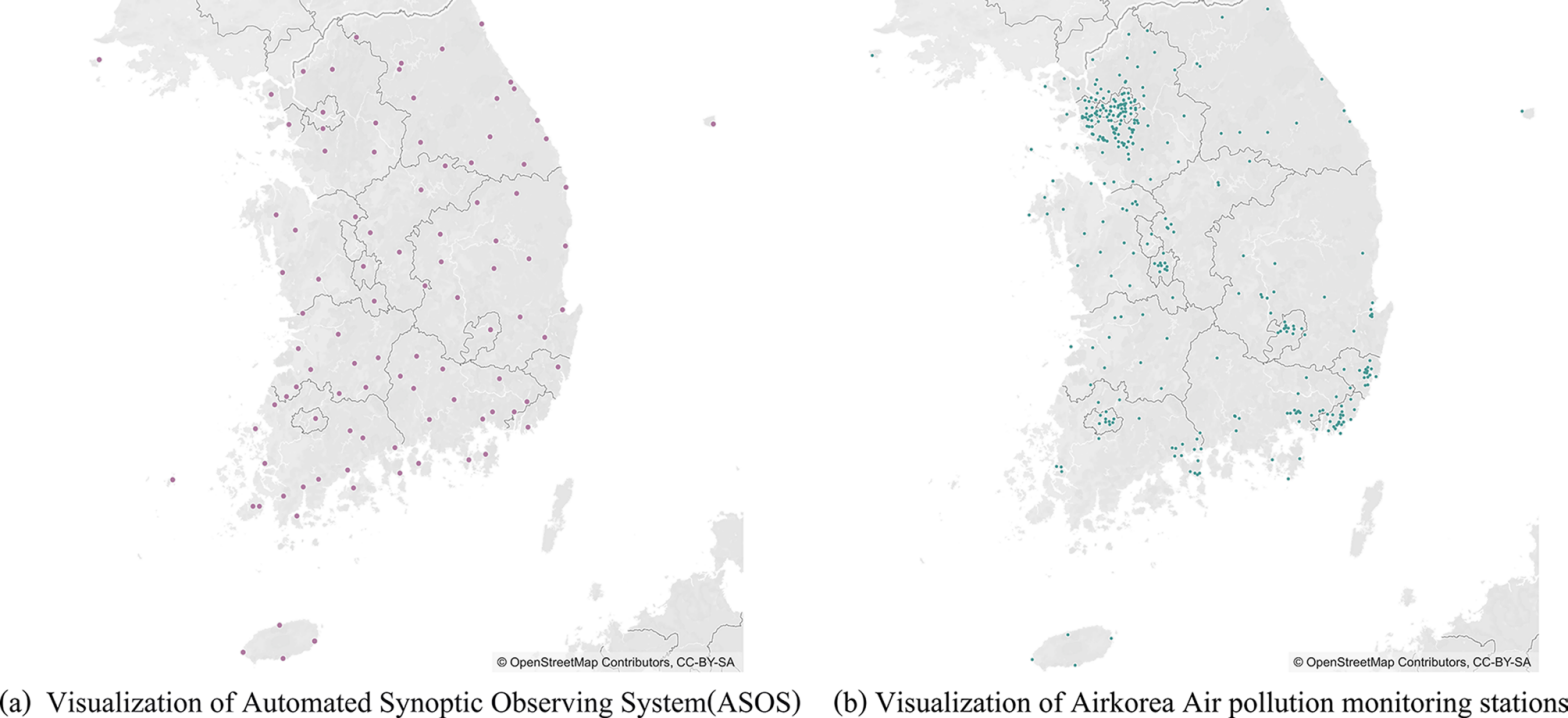
Visualization of air quality and weather monitoring stations. Map image is obtained from OpenStreetMap (openstreetmap.org) and licensed under CC-BY-SA (https://www.openstreetmap.org/copyright).

Air quality and weather data collected in South Korea were used in this study. The air quality data were collected hourly at air pollution monitoring stations (432 in 2018 and 484 in 2019) operated by Korea Environment Corporation for SO_2_, CO, O_3_, NO_2_, PM10, and PM2.5. The collection took place from 01:00 on January 1, 2018 to 24:00 on December 31, 2019. Weather data including temperature, precipitation, wind direction, and wind speed were collected at 102 Automated Synoptic Observing System stations provided by the Korea Meteorological Administration. The weather data were collected during the same collection period of the air quality data. The locations of the monitoring stations are shown in Fig. 2.

However, some monitoring stations contained missing data due to closed or newly established stations. Consequently, we used data from 243 air quality monitoring stations and 94 weather monitoring stations, which operated continually for 2 years. The percentage of missing air quality data during this period was approximately 2.882%, 2.931%, 2.814%, 3.213%, 4.182%, and 6.210% for SO_2_, CO, O_3_, NO_2_, PM10, and PM2.5, respectively. The percentage of missing weather data was approximately 0.089%, 0.211%, 0.233%, and 90.880% for temperature, wind speed, wind direction, and precipitation, respectively. Missing values were interpolated by IDW. However, the precipitation data were treated as missing if the measured value of precipitation was less than 0.05 mm or if the precipitation was not measured. As these two cases were not distinguishable, all the missing precipitation data were replaced with zeros based on the assumption that the readings were less than 0.05 mm. The wind direction represents the direction of the wind from 0° to 360°, and we converted the wind direction to X,Y coordinate data. The total 11 variables data prepared through this process was used as initial input data for the ICNN prediction model. The descriptive statistics were calculated after interpolation, as shown in Table 1.

**Table 1:** Descriptive statistics of data after interpolation of missing data.

Variables	Observations	Min	Mean	Max	Standard deviation
PM10 ( µg /m^3^)	4,257,360	0.000	41.940	565.000	28.462
PM2.5 ( µg /m^3^)	4,257,360	0.000	23.060	262.000	18.346
SO_2_ (ppm)	4,257,360	0.000	0.004	0.376	0.003
CO (ppm)	4,257,360	0.000	0.474	8.900	0.227
O_3_ (ppm)	4,257,360	0.000	0.039	11.561	0.284
NO_2_ (ppm)	4,257,360	0.000	0.213	0.176	0.015
Temperature (℃)	4,257,360	− 22.500	13.510	39.700	10.587
Precipitation (mm)	4,257,360	0.000	0.138	94.000	1.018
Wind speed (m/s)	4,257,360	0.000	1.931	27.000	1.330
X-coordinate of wind direction	4,257,360	− 0.006	− 0.001	0.006	0.004
Y-coordinate of wind direction	4,257,360	− 0.006	− 0.001	0.006	0.004

### Baseline

**Figure 3:**
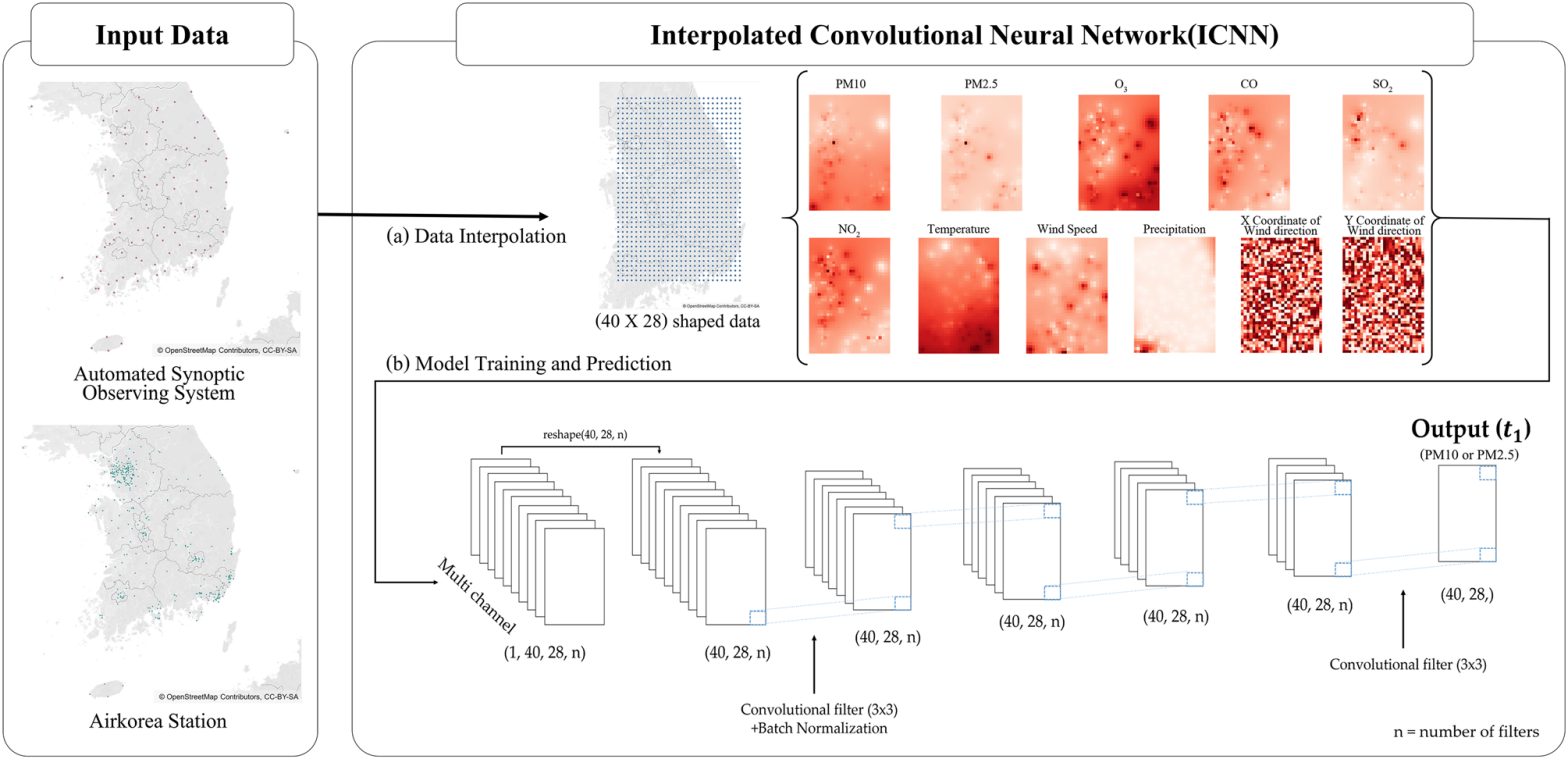
Overall flow of the ICNN prediction model. Map image is obtained from OpenStreetMap (openstreetmap.org) and licensed under CC-BY-SA (https://www.openstreetmap.org/copyright).

The ICNN prediction model performs two functions: interpolating the spatially imbalanced data into equal distance data and predicting the transformed data after training through the CNN model. The overall flow of the ICNN prediction model is illustrated in Fig. 3.

#### (a) Data interpolation

The input data of the ICNN prediction model are numerical data including six air quality and five weather variables collected at the monitoring stations. Before the interpolation, we set the grid size where the data would be filled. First, we drew a rectangle based on the monitoring stations located at the easternmost, westernmost, northernmost, and southernmost points. The length (North to South) and width (East to West) dimensions of the rectangle were found to be approximately 403.6 km and 280.84 km, respectively. The rectangle was then partitioned into 40 rows and 28 columns for a total of 1120 points, based on the coordinates. The location of the grid’s upper left corner was denoted (1,1) and the location of the lower right corner was (40,28). Each grid cell had a width of 10.03 km and height of 10.09 km. The 11 air quality and weather data used as input data were interpolated separately to fit the empty (40 × 28) sized grid. The grid data generated by IDW interpolation were then used as input data in the next step in the ICNN prediction model.

#### (b) Model training and prediction

The CNN model that performs training and prediction in the ICNN prediction model was configured with 11 variables as a multi-channel matrix, which was used as the input data. The input data were split into train:validation:test data in a 6:2:2 ratio. We then set up the CNN model separately for PM10 and PM2.5 to predict levels over the next hour. The CNN model of the ICNN prediction model trains and predicts a multi-channel matrix like an image through various layers. In layer 1, a (12 × 1 × 1) filter was employed to reduce the dimensions of the time step included in the input data. In layer 2, the dimensions of the time step, which were reduced and only nominally indicated, were removed through reshaping. A convolution operation was then performed using a (3 × 3) filter from layers 3 to 7. The final output consisted of a single-channel, (40 × 28) image. Subsequently, the weight of the filter was optimized by comparing the final output with the actual data. To optimize the fitness of the ICNN prediction model, we determined through comparison the optimal combination of parameters. For this step, we used ReLU and scaled exponential linear unit as the activation functions. These functions are robust to gradient vanishing, which has been identified as a problem in neural networks. In addition, among the optimizers for weights in neural networks, we used Adadelta, Adam, and SGD optimization algorithms. After multiple attempts, we selected ReLU and Adam as optimal learning parameters, a batch size of 512, epoch of 2000, mean squared error for loss function, and early stopping to prevent overfitting. Furthermore, we enhanced the model’s performance by using batch normalization. We used the OLS and LSTM models as comparison groups for the ICNN prediction model. The comparison models were configured to predict a total of 1120 points (40 × 28 = 1120), which is the same as the output of the ICNN prediction model.

### Evaluation metric

#### R-squared

R-squared is an indicator used to evaluate the explanatory power of spatial and temporal prediction models^45^. In this study, the R-squared value was derived by performing an ordinary least squares regression using the PM10 and PM2.5 concentration levels measured and predicted by the ICNN prediction model as independent variables and the hourly measured, actual PM10 and PM2.5 concentrations as dependent variables. The derived R-squared value was used as the explanatory power indicator of the prediction model.

#### Root mean squared error (RMSE)

The RMSE is the mean error between the predicted value and the measured value. It is one of the most frequently used evaluation indicators to represent the general performance of prediction models^19,21,26,45^. The closer to zero the RMSE value, the better the prediction model. In this study, the performance of the prediction model was evaluated by calculating the mean error between the air quality variable concentration predicted by the ICNN prediction model and the collected data.

#### Verification of high concentration

The fine dust(PM10) high concentration criterion of 80 µg or higher and the ultrafine dust(PM2.5) high concentration criterion of 35 µg were classified as high concentrations as per the Republic of Korea Ministry of Environment. The following evaluation indicators were used for evaluating high PM10 and PM2.5 concentrations^13,46,47^: probability of detection (POD), false alarm rate (FAR), true skill score (TSS), and critical success index (CSI).

In Table 1, the contingency table needed for using each indicator in binary classification^48^ is presented. If the forecast is accurate, the possible cases are "Hit" and "Correct rejection"; if the forecast is not accurate, the possible cases are "False alarm" and "Miss." The overall accuracy of the prediction model is good if the "Hits" and "Correct rejections" are predominant, with a few "False alarms" and "Misses." The equations for POD, FAR, CSI, and TSS, using the parameters shown in Table 2, are as follows:

**Table 2:** Contingency table.

		Observed	
		Yes	No
Forecast	Yes	Hit (a)	False alarm (b)
	No	Miss (c)	Correct rejection (d)

1 $$POD= \frac{a}{a+c}$$

2 $$FAR= \frac{b}{b+d}$$

3 $$TSS=\left(\frac{a}{a+c}\right)-\frac{b}{b+d}$$

4 $$CSI= \frac{a}{a+b+c}$$

POD, similar to “recall,” is a test method for measuring the ratio of accurate forecasts of events, which ranges between 0 and 1, with a POD value closer to 1 indicating a better prediction model. The POD reacts sensitively to events but does not consider the cases of no event. Therefore, the performance of POD can be artificially improved by excessively generating “Yes”.

FAR, similar to “1-precision,” is a test method for measuring the ratio of false alarms: incidents of predicting that there is an event when there is no event. The FAR also ranges between 0 and 1, with a better prediction indicated by a FAR closer to zero. In contrast to POD, FAR reacts sensitively to "False alarm," and its performance can be artificially improved by excessively generating “No.” Thus, POD and FAR are usually tested together.

TSS is used when the prediction model handles POD and FAR. Ideally, TSS is determined by the ability to distinguish between “Yes” and “No” cases. Therefore, TSS can be said to be an indicator that evaluates the artificial-performance-improving POD and FAR. The TSS ranges between -1 and 1, with 1 indicating a perfect forecast and 0 defining the standard forecast, negative value indicating a below standard forecast. The CSI considers "False alarm" and "Miss" together while excluding "Correct rejection," and it sensitively reacts to “Hit.” Thus, it is used as a performance measurement criterion for rare events. The CSI varies between 0 and 1, with 1 indicating a better prediction model.

### Experimental setup

Timestep 1 and 12 models were implemented using Intel(R) Core i9-7900X CPU @ 3.30 Ghz and four Nvidia GTX 1080 Ti. Timestep 24 models were implemented using Intel(R) Xeon(R) Silver 4110 CPU @ 2.10 GHz and two Nvidia TITAN RTX. The ICNN prediction model and LSTM model were trained and implemented on Tensorflow-GPU 1.15.0 and keras (v. 2.2.5). The OLS model was trained and implemented using R (v. 3.4.3).

## Results

This paper proposed an ICNN prediction model that predicts particulate matters by using interpolated spatially uniform data. We designed a long-term prediction model for forecasting PM10 and PM2.5 for up to 24 h. The time lag was set to 1, 2, 4, 6, 12, and 24 h. In addition, the time step for the training data was set to 1, 12, or 24 h. Each PM10 and PM2.5 variable was used as the target data, and the short-, mid-, and long-term forecasts were performed independently for three time steps, by comparing the results.

**Figure 4:**
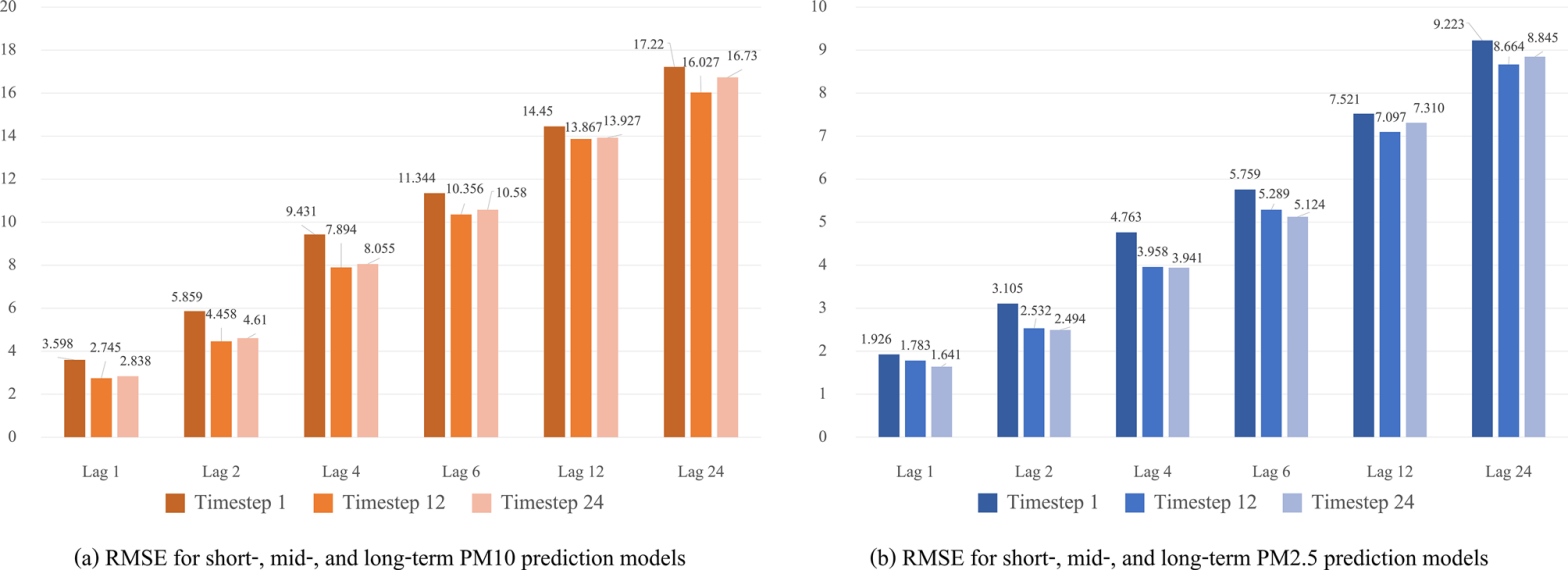
RMSE for short-, mid-, and long-term prediction models.

The forecast results of a total of 36 model runs, performed for three time steps and six time lags are shown in Fig. 4. The top and bottom graphs show the PM10 and PM2.5 long-term prediction model results, respectively.

In 36 models, the RMSE value for time lag 1 was the smallest, and the RMSE value increased as the time lag increased. However, the RMSE values of all models were lower than the standard deviation of PM10 and PM2.5. This result confirmed that all 36 models showed adequate prediction results. It should be noted that the one-hour forecasting (lag 1) model in PM10 and PM2.5, among 36 models, performed the best.

The results of the long-term prediction model of PM10 are shown in Fig. 4a, which demonstrates that the RMSE of the model with a 12-h time step showed relatively good performance in all time lags. The results of the long-term prediction model of PM2.5 are shown in Fig. 4b, which demonstrates that the RMSE of the model with a 24-h time step showed relatively good performance, except when the time lag was 12 and 24 h. This result confirmed that appropriate forecast results can be derived at every time lag when the time step is 12 h or 24 h.

Table 3 summarizes the RMSE and R-squared values for the PM10 and PM2.5 air quality parameters as generated by the ICNN prediction model, which performed best in a 1-h forecast compared with the other models in the same condition. The one-hour forecast of the PM10 and PM2.5 concentrations in the ICNN prediction model showed R-squared values higher than 0.97 and an RMSE of 15.619–15.721% of the standard deviation. The 1-h forecast of the PM10 and PM2.5 concentrations in the LSTM model showed R-squared values higher than 0.93 and an RMSE of 24.343–25.115% of the standard deviation. And the one-hour forecast of the PM10 and PM2.5 concentrations in the OLS model showed R-squared values higher than 0.71 and an RMSE of 26.029–28.700% of the standard deviation. These results confirm that more accurate and reliable conclusions can be obtained by using the ICNN prediction model than other existing methods.

**Table 3:** RMSE and R-squared values for the one-hour forecast by ICNN and comparative models.

Model	Variable	Best result timestep	Standard deviation	RMSE	R-squared
ICNN	PM10	12	17.460	2.745	0.975
LSTM				4.385	0.934
OLS				5.011	0.712
ICNN	PM2.5	24	10.500	1.640	0.976
LSTM				2.556	0.936
OLS				2.733	0.740

In Table 4, the POD, FAR, CSI, and TSS for classifying and forecasting high concentration PM10 and PM2.5 are shown. The evaluation indicators show that the ratio of accurate predictions of high concentrations is high, and the ratio of erroneous predictions of high concentrations is low. This implies that the prediction model proposed in this study is very reliable, because it correctly predicts high and low concentrations. The CSI is sensitive to correct forecasts of high concentration, and the CSI values presented here indicate that the high-concentration events for PM10 and PM2.5 are generally forecasted correctly.

**Table 4:** Evaluation of forecasts for high concentration PM10/PM2.5 using ICNN.

Variable	POD	FAR	TSS	CSI
PM10	0.926	0.001	0.925	0.876
PM2.5	0.901	0.004	0.897	0.854

## Discussion

In this study, we developed an ICNN, which can effectively perform spatio-temporal prediction, and used it for forecasting air quality in South Korea. Data on PM10 and PM2.5 variable concentrations were collected at multiple monitoring station locations, interpolated, and combined with a CNN model to create the ICNN model for air quality prediction. The PM10 and PM2.5 prediction models showed high forecast accuracy and explanatory power, and the possibility of future improvement was verified by introducing a long-term prediction model for these variables.

The proposed ICNN prediction model has the following advantages:

First, the ICNN prediction model can be expected to produce high PM prediction accuracy by learning spatio-temporal information from big data. In the case of the previous prediction models, it is difficult to learn spatio-temporal information effectively. The ICNN prediction model directly handles spatio-temporal information by using interpolation, which can convert non-uniform data to uniform data, and the CNN, which can efficiently use spatial information. Furthermore, the model shows high numerical prediction performance, indicated by the high R-squared values of 0.975 and 0.976 for PM10 and PM2.5, respectively, and the high corresponding RMSE values of 2.745 and 1.640. In addition, when concentration events higher than 80 µg for PM10 and 35 µg for PM2.5 were forecast, the evaluation indicators were calculated as follows: the POD scored 0.926 and 0.901, respectively; the FAR scored 0.001 and 0.004, respectively; TSSs were 0.925 and 0.897, respectively; CSIs were 0.876 and 0.854. Based on these results, this model clearly performs well in classifying high concentration events.

Second, we can learn effectively with the CNN model by transforming the non-uniform data that was measured by monitoring stations in different locations into spatially uniform data. The existing air quality monitoring models have shown limitations in measuring and forecasting PM in suburban areas, due to the monitoring stations being concentrated in highly populated downtown areas. However, the ICNN prediction model proposed in this paper can forecast PM in suburban areas as well, by using the air quality data from areas with actual monitoring stations and interpolation to predict the air quality in unmonitored areas. Furthermore, this ICNN prediction model can predict PM for all areas simultaneously by many to many prediction models.

However, our study has a limitation. Overseas-generated factors affecting the Korea Peninsula were not considered in this study. For example, the air pollution caused by China is wind-borne over Korea^49,50^, but this study did not consider its impact on air quality in South Korea.

Despite its limitation, the ICNN prediction model we propose here can be a useful tool for predicting pollution like the concentrations of air pollutants for longer collection periods and larger areas containing evenly spaced monitoring stations. Furthermore, although the proposed model showed the possibility of long-term predictions, areas for future improvement in learning past times were identified.

In conclusion, the proposed ICNN prediction model can be an effective forecasting tool in various environmental areas, including air quality, and it also presents a new perspective in the prediction field.
